# Suicide risk in people with post-traumatic stress disorder: A cohort study of 3.1 million people in Sweden

**DOI:** 10.1016/j.jad.2020.10.009

**Published:** 2021-01-15

**Authors:** Verity Fox, Christina Dalman, Henrik Dal, Anna-Clara Hollander, James B. Kirkbride, Alexandra Pitman

**Affiliations:** a Division of Psychiatry, UCL, London, W1T 7NF, United Kingdom; b Department of Global Public Health, Karolinska Institutet, Sweden; c Centre for Epidemiology and Social Medicine, The Region Stockholm, Sweden; d Camden and Islington NHS Foundation Trust, London, NW1 0PE, United Kingdom

**Keywords:** Suicide, Post-traumatic stress disorder, PTSD, Epidemiology, Cohort study

## Abstract

**Background:**

It is unclear whether post-traumatic stress disorder [PTSD] is associated with suicide risk in the general population, whether this differs by sex, or what the population impact of PTSD is for suicide.

**Methods:**

We constructed a nationwide cohort of all people living in Sweden, born 1973–1997, followed from their 14th birthday (or immigration, if later) until suicide, other death, emigration or 31 December 2016. We used Cox proportional hazards regression to estimate hazard ratios [HR], and calculated the population impact of PTSD on suicide. We included sensitivity analyses to explore effects of outcome and exposure definitions, and to account for potential competing risks.

**Results:**

Of 3,177,706 participants, 22,361 (0•7%) were diagnosed with PTSD, and 6,319 (0•2%) died by suicide over 49•2 million person-years. Compared with women and men without PTSD, suicide rates were 6•74 (95%CI: 5•61–8•09) and 3•96 (95%CI: 3•12–5•03) times higher in those with PTSD, respectively, after sociodemographic adjustment. Suicide rates remained elevated in women (HR: 2•61; 95%CI: 2•16–3•14) and men (HR: 1•67; 95%CI: 1•31–2•12) after adjustment for previous psychiatric conditions; attenuation was driven by previous non-fatal suicide attempts. Findings were insensitive to definitions or competing risks. If causal, 1•6% (95%CI: 1•2–2•1) of general population suicides could be attributed to PTSD, and up to 53.7% (95%CI: 46.1–60.2) in people with PTSD.

**Limitations:**

Residual confounding remains possible due to depressive and anxiety disorders diagnosed in primary care but unrecorded in these registers.

**Conclusions:**

Clinical guidelines for the management of people with PTSD should recognise increased suicide risks.

## Highlights

• PTSD is a risk factor for suicide, particularly in women.
• Risk is primarily attenuated by suicide attempts pre-PTSD diagnosis.
• PTSD accounts for 0•6% of suicides in men and 3•5% in women.
• Up to 54% of suicides in people with PTSD are attributable to PTSD.

## 1 Introduction

Post-traumatic stress disorder [PTSD] is a psychiatric diagnosis describing a prolonged or delayed response to a traumatic situation or event(s) of a threatening or catastrophic nature (World Health Organisation, 1992). Symptoms include repeatedly reliving the experience, in the form of intrusive memories known as “flashbacks” or repeated dreams, experiencing significant distress when exposed to a situation similar to or associated with the traumatic experience, avoidance of such situations, inability to recall aspects of the trauma, or persistent symptoms of psychological arousal not present before the trauma. A recent World Health Organisation [WHO] study in 24 countries estimated that lifetime PTSD prevalence varied from 2•1% in lower low-middle income countries to 5•0% in high-income countries (Koenen et al., 2017) including Sweden (5•6% (Frans et al., 2005)). Lifetime prevalence also varies by gender, and is consistently reported as being twice as high in women than men, with one US study placing estimates at 10•4% and 5•0%, respectively (Kessler et al., 1995).

Such variation in prevalence may arise due to differential exposure to traumatic factors, differences in internalising and externalising such experiences, differences in recovery and survival following PTSD or differences in diagnostic practice in different settings. For example, PTSD is a risk factor for subsequent suicidal ideation and attempt (Bentley et al., 2016), and has been linked to death by suicide (Krysinska and Lester, 2010), although results from these systematic reviews and meta-analyses are inconsistent. This may be attributable to methodological issues: most studies have been prevalence-based, conducted in veteran or military samples, or have sometimes inappropriately adjusted for comorbid mental health conditions on the causal pathway between PTSD and suicide. Only two population-based studies have examined the association between PTSD and death by suicide, both conducted using Danish register data (Gradus et al., 2010, 2015). The first, a case-control study of all suicide deaths in Denmark between 1994 and 2006, found that the odds of death by suicide was 5.3 times higher in those with a diagnosis of PTSD compared with those without, after controlling for psychiatric and other demographic variables (Gradus et al., 2010). A second study, following a Danish population cohort between 1995 and 2011, found that suicide rates were 13 times greater in those diagnosed with PTSD, adjusting for confounders as above (Gradus et al., 2015). Although the largest of these studies (Gradus et al., 2015) included over 500,000 participants, given the absolute rarity of both PTSD and death by suicide, it could only include 32 participants with both the exposure and outcome, limiting the ability to detect variation by other characteristics such as sex, or control for all previous major psychiatric conditions or previous non-fatal suicide attempts.

Further studies, in larger population-based samples, are required to clarify such issues. Gender differences in the association between PTSD and death by suicide might be expected because women are at increased risk of PTSD (Atwoli et al., 2015; Ditlevsen and Elklit, 2012), but in non-clinical populations are less likely to die by suicide than men in many high income countries (Värnik, 2012). Accordingly, a study of US veterans reported that suicide rates associated with PTSD were greater for women than men (Ilgen et al., 2010), but this has yet to be established in general population samples. Understanding gender-specific risk factors is important, particularly in young and middle-aged groups, who are the current focus of public health concerns about suicide (Pitman et al., 2012).

We sought to investigate whether a PTSD diagnosis predicted death by suicide in a large, nationwide cohort of people living in Sweden. We also investigated whether this association differed between men and women. To understand the potential impact of PTSD on death by suicide at the population level, we estimated the population attributable risk faction and the attributable fraction in those with a PTSD diagnosis in our cohort. We hypothesised that individuals with a diagnosis of PTSD would be at increased risk of death by suicide compared to those without a PTSD diagnosis, and that the association would vary by gender with greater relative risks of suicide following PTSD for women, given previous evidence (Ilgen et al., 2010). We examined the sensitivity of our findings to different exposure and outcome definitions and potential competing risks.

## 2 Methods

### 2.1 Design & setting

We conducted a cohort study to identify individuals in the Swedish population diagnosed with PTSD after 14 years old, compared with individuals without a PTSD diagnosis. Our cohort included all individuals born between 1973 and 1997, and living in Sweden on or after their 14th birthday, including people who immigrated to Sweden up to the end of 2011 (see Supplemental file). Participants were followed from their 14th birthday or, if later, date of immigration to Sweden, until exit from the cohort (either death, emigration, or the end of follow-up: 31 December 2016).

### 2.2 Ethical approval

We were granted access to de-identified linked data from Statistics Sweden after approval from the Stockholm Regional Ethical Review Board (number 2010/1185–31/5).

### 2.3 Outcome

Our primary outcome was suicide death, as recorded in the Cause of Death Register, which also includes deaths of Swedish citizens who died abroad if they had left Sweden temporarily and died during this time (Brooke et al., 2017). In Sweden, causes of death were recorded using the International Classification of Diseases, ninth revision [ICD-9] from 1987 to 1996, and the tenth revision [ICD-10], since 1997. We included participants with a cause of death by suicide (ICD-9: E950–959; ICD-10: X60–84) or death by undetermined intent (ICD-9: E980–989; ICD-10: Y10-Y34) as our primary outcome, consistent with previous studies (A.C. Hollander et al., 2019) and evidence that restriction to cases with a suicide verdict leads to an underestimation of overall suicide rates (Carroll et al., 2012).

### 2.4 Exposure

Our exposure was a binary variable denoting a clinical diagnosis of PTSD before death by suicide during the follow-up period, as recorded in the National Patient Register (Ludvigsson et al., 2011), and previously validated for research (A.C. Hollander et al., 2019). This register recorded all inpatient visits for psychiatric conditions since 1987 and outpatient visits since 2001 to public and private secondary healthcare facilities and emergency departments. National inpatient coverage has been 100% complete since 1987, and complete for outpatient settings since 2006. Between 2001–2005, national outpatient coverage was approximately 80% complete (Ludvigsson et al., 2011). Since 1997, PTSD was diagnosed in Sweden using the ICD-10 code F43•2. From 1987–1996, the Swedish version of ICD-9 did not include a specific PTSD code (although one was introduced to the main ICD-9 edition, as 309•81). Instead, we defined PTSD using the Swedish ICD-9 code 309B “delayed crisis reaction”, excluding the codes “acute stress reactions” (309A) and “crisis reactions, not otherwise specified” (309X), consistent with previous work (Song et al., 2018). We conducted a sensitivity analysis around this definition (see below).

### 2.5 Confounders

We selected the following sociodemographic and clinical variables as potential confounders, based on clinical experience and the literature: natal sex, attained age, birth region (based on major migrant flows to Sweden (Hollander et al., 2016): Sweden, Finland, other Nordic countries, Western and Southern Europe, Eastern Europe & Russia, Asia & Oceania, Middle East & North Africa, sub-Saharan Africa, North America and South America), neighbourhood deprivation level and population density, previous non-fatal suicide attempt (coded using ICD-9 and ICD-10 codes as above for suicide, but relating to hospitalisation for non-fatal suicide attempt; see Supplemental file), previous diagnosis for major psychiatric conditions (major depressive or anxiety disorders, non-affective psychotic disorders, bipolar disorders) and parental history of severe mental illness (see Supplemental file).

### 2.6 Statistical analysis

First, we reported basic descriptive statistics for the cohort and examined mortality rates for suicide within the population. Using Cox proportional hazard models, we then examined whether suicide mortality rates differed by PTSD status, by estimating hazard ratios [HR] and 95% confidence intervals [95% CI]. Since suicide risk varies by age (Shah, 2012), we treated age as a time-varying covariate, using Lexis expansion to stratify participants into multiple age observations (14–19, 20–24, 25–29, 30–34, 35–39, 40–44 years old). We ran three separate models: a univariable model, a multivariable model adjusted for all sociodemographic variables simultaneously, and a fully-adjusted model with further adjustment for clinical variables. To test for an interaction between PTSD and sex, we added an interaction term to our fully-adjusted model, compared to a model without the interaction via likelihood ratio test. We presented stratified results where appropriate. We calculated population attributable risk fractions [PAF] and the attributable fraction in the exposed [AFe], with 95% CIs (see Supplemental file), to ascertain the proportion of suicides in our population at-risk and those exposed to PTSD, respectively, which could be attributed to PTSD, assuming causality. We checked models for violation of proportional hazards using log-log plots and a Schoenfeld residuals test. Given a small amount of missing data (0•5%), our analyses were based on complete case data. For all analyses we used Stata 15 (StataCorp, 2017).

### 2.7 Sensitivity analyses

We conducted four sensitivity analyses. First, we examined whether using a broader definition of ICD-9 PTSD (“acute stress reactions” (309A) and “crisis reactions, not otherwise specified” (309X) included) altered any observed associations between PTSD and death by suicide. Second, we re-ran our models to determine whether our results differed for death by suicide versus undetermined intent. Third, we re-ran our models, excluding people diagnosed with PTSD as outpatients between 2001 and 2005 when national outpatient coverage was incomplete, to assess for any potential bias this introduced in our main results. Finally, we remodelled our data using a Fine and gray (Fine and Gray, 1999) competing risks regression to inspect the sensitivity of our results to the possibility that other deaths or emigration could have acted as a competing risk for suicide (see Supplemental file).

## 3 Results

From our initial cohort of 3194,141 individuals, 3177,706 (99•5%) with complete data were included in the analysis, contributing to over 49•2 million person years of follow up. We excluded 16,435 (0•5%) participants with missing data on model covariates (Supplemental file, Table 2), who were more likely to be men, younger, born outside of Sweden, have a history of major psychiatric disorders including PTSD (all *p*<0•0001; Supplemental file, Table 2), but not more likely to die by suicide (*p* = 0•13) or all-cause mortality (*p* = 0•45).

In this cohort, 22,361 (0•7%) were diagnosed with PTSD, of whom 192 (0•9%) died by suicide during follow up (Table 1). PTSD diagnoses were more common in migrants (1•1% versus 0•6%; Χ^2^ test on 1 degree of freedom: 494•0; *p*<0•0001) than the Swedish-born population. Median age at suicide was 25•2 years old (interquartile range [IQR]: 21•2–30•6) in those without PTSD, but was significantly higher in people with PTSD (28•2 years; IQR: 23•9–33•3; Mann-Whitney *z*=−5•3; *p*<0•0001). Median age at PTSD diagnosis was 26•1 (IQR: 20•6–32•0) years, with a subsequent median time to suicide of 2•4 years (IQR: 0•9–5•0). In people without PTSD, suicide was more common in men (72•9% vs• 27•1%), Swedish-born participants, those in more deprived areas at cohort entry, and those with a previous personal or parental history of mental health problems. Similar patterns were observed in those with PTSD, except suicide was more common in females than males (64•1% vs• 35•9%). Full cohort characteristics are presented in Table 1.

**Table 1:** Cohort characteristics by PTSD diagnosis status.

	PTSD (*N* = 22,361; 0•7%)		No PTSD (*N* = 3155,345; 99•3%)	
	**Person-years^1^ n (%)**	**Suicide n (%)**	**Person-years^1^ n (%)**	**Suicide cases n (%)**
Total	356,172 (100)	192 (0•9)^2^	48,721,903 (100)	6127 (0•2)^2^
*Sociodemographic factors*				
Age at cohort exit [years: median (IQR)]	31•9 (26•0–38•0)	28•2 (23•9–33•3)	30•5 (24•9–37•1)	25•2 (21•2–30•6)
Natal sex				
Male	102,645 (28•8)	69 (35•9)	25,058,232 (51•4)	4464 (72•9)
Female	253,527 (71•2)	123 (64•1)	23,663,671 (48•6)	1663 (27•1)
Country of origin				
Sweden	251,747 (70•7)	147 (76•2)	41,759,042 (85•7)	5438 (88•8)
Finland	1497 (0•4)	–	186,727 (0•4)	56 (0•9)
Other Nordic countries	1657 (0•5)	4 (2•1)	264,575 (0•5)	26 (0•4)
Western & Southern Europe	2663 (0•7)	1 (0•5)	453,140 (0•9)	30 (0•5)
Eastern Europe & Russia	27,762 (7•8)	13 (6•8)	1707,230 (3•5)	141 (2•3)
Asia & Oceania	14,328 (4)	10 (5•2)	1243,530 (2•6)	143 (2•3)
Middle East & North Africa	39,623 (11•1)	10 (5•2)	1844,773 (3•8)	144 (2•4)
Sub-Saharan Africa	8039 (2•3)	5 (2•6)	635,435 (1•3)	55 (0•9)
North America	1621 (0•5)	–	164,200 (0•3)	18 (0•3)
South America	7235 (2)	2 (1•0)	463,251 (1)	76 (1•2)
*Area level characteristics*				
Population density^3^				
1 (Most rural)	62,705 (17•6)	36 (18•8)	10,471,947 (21•5)	1366 (22•3)
2	62,502 (17•5)	33 (17•2)	10,278,574 (21•1)	1263 (20•6)
3	63,835 (17•9)	38 (19•8)	10,084,757 (20•7)	1192 (19•5)
4	78,566 (22•1)	42 (21•9)	9625,205 (19•8)	1216 (19•9)
5 (Most urban)	88,564 (24•9)	43 (22•4)	8261,420 (17)	1090 (17•8)
Deprivation index				
1 (least deprived)	51,607 (14•5)	26 (13•5)	10,351,971 (21•2)	1097 (17•9)
2	55,856 (15•7)	31 (16•2)	9956,957 (20•4)	1142 (18•6)
3	67,708 (19)	35 (18•2)	10,144,911 (20•8)	1273 (20•8)
4	81,262 (22•8)	53 (27•6)	9853,343 (20•2)	1415 (23•1)
5 (most deprived)	99,739 (28)	47 (24•5)	8414,721 (17•3)	1200 (19•6)
*Clinical characteristics^4^*				
Previous major depression or anxiety disorders	50,234 (14•1)	70 (36•5)	4962,487 (10•2)	2370 (38•7)
Previous bipolar disorder diagnosis	20,676 (5•8)	21 (10•9)	496,721 (1)	340 (5•6)
Previous non-affective psychosis diagnosis	16,453 (4•6)	19 (9•9)	426,068 (0•9)	633 (10•3)
Previous non-fatal suicide attempts	82,387 (23•1)	108 (56•3)	1630,277 (3•3)	1602 (26.1)
Parental history of SMI^5^	23,996 (6•7)	27 (14•1)	1625,511 (3•3)	550 (9•0)

PTSD: post-traumatic stress disorder; IQR: interquartile range; CMD: common mental disorder; SMI: severe mental illness.

^1^ Rounded to the nearest integer.

^2^ Percentage of those with or without PTSD.

^3^ People per km^2^, quintiles.

^4^ Diagnosis made either before PTSD (in those with PTSD) or before cohort exit (in those without PTSD).

^5^ Severe mental illness in biological parents.

In the total Swedish population the crude mortality rate for death by suicide was 12•9 (95% CI: 12•6–13•2) per 100,000 person years (Table 2), rising to 53•9 (95% CI: 46•8–62•1) in those with a PTSD diagnosis, although this was higher for men (67•2; 95% CI: 53•1–85•1) than women (48•5; 95% CI: 40•6–57•9; *p* = 0•02). The overall rate difference for those with and without a PTSD diagnosis was 41•3 (95% CI: 33•7–49•0) per 100,000 person years.

**Table 2:** Incidence rates and hazard ratios for suicide by PTSD status.

	Crude mortality rate^1^		Hazard ratios (95% CI)		
	**Rate**	**95% CI**	**Unadjusted**	**Adjustment 1^2^**	**Adjustment 2^3^**
Total	12•9	(12•6–13•2)			
PTSD					
No	12•6	(12•3–12•9)	1	1	1
Yes	53•9	(46•8–62•1)	4•28 (3•70–4•94)	5•36 (4•64–6•20)	2•16 (1•86–2•50)
Men	67•2	(53•1–85•1)	3•81 (3•00–4•83)	3•96 (3•12–5•03)	1•67 (1•31–2•12)
Women	48•5	(40•6–57•9)	6•85 (5•70–8•23)	6•74 (5•61–8•09)	2•61 (2•16–3•14)
LRT test *Χ*^2^ for interaction (df); p-value			15•3 (1); 0•0001	12•5 (1); 0•0004	8•8 (1); 0•003
Broadly-defined PTSD^4^					
No	12•5	(12•2–12•8)	1	1	1
Yes	62•5	(55•3–70•6)	4•93 (4•35–5•58)	6•11 (5•38–6•93)	2•50 (2•19–2•84)
Men	89•3	(74–107•8)	5•01 (4•14–6•06)	5•14 (4•25–6•23)	2•23 (1•84–2•71)
Women	51•4	(43•8–60•3)	7•26 (6•14–8•58	7•10 (6•01–8•39)	2•74 (2•32–3•25)
LRT test Χ^2^ for interaction (df); p-value			8•3 (1); 0•004	6•3 (1); 0•01	2•6 (1); 0•11

LRT: likelihood ratio test; df: degrees of freedom; PTSD: post-traumatic stress disorder; 95% CI: 95% confidence intervals.

^1^ per 100,000 person-years.

^2^ Adjusted for current age, natal sex (except in stratified analyses), country of origin, population density and deprivation.

^3^ Adjusted for variables listed in Adjustment 1, previous major depression or anxiety disorders, bipolar disorder, non-affective psychotic disorder, previous non-fatal suicide attempts and parental history of severe mental illness.

^4^ See first sensitivity analysis for definition.

**Fig. 1:**
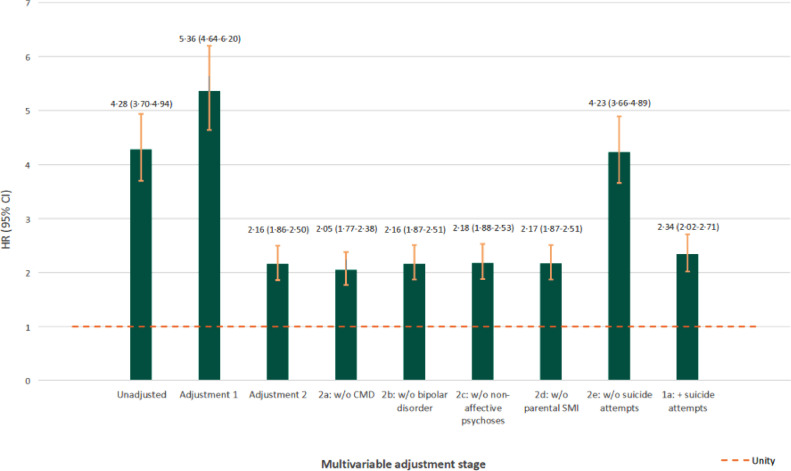
Examination of confounding effect of clinical variables in Cox regression models. Legend: Hazard ratios [HR] and 95% confidence intervals [95% CI] for risk of suicide associated with a diagnosis of post-traumatic stress disorder [PTSD], with various stages of adjustment during multivariable modelling. Results for unadjusted and adjusted models 1 and 2 are presented in Table 2. ***Adjustment 1*** includes control for current age, natal sex, country of origin, population density and deprivation. ***Adjustment 2*** includes additional control for previous diagnoses of psychiatric conditions before a PTSD diagnosis and parental severe mental illness [SMI], and shows substantial attenuation that can be attributed to one or more of these conditions. ***Adjustments 2a-2e*** show the risk of suicide associated with PTSD after adjusting for all variables in adjusted model 2, except the psychiatric condition stated. Models 2a-2d show that a diagnosis of major depression or anxiety disorder, bipolar disorder or non-affective psychotic disorder prior to a PTSD diagnosis or parental history of SMI do not explain attenuated suicide risk between adjusted models 1 and 2. Model 2e, however, shows that without control for previous non-fatal suicide attempts, the association between PTSD and suicide is stronger (HR: 4•23; 95% CI: 3•66–4•89), suggesting that previous non-fatal suicide attempts are an important confounder of the association between PTSD and later suicide risk. ***Adjustment 1a*** shows adjusted model 1 with additional control for previous non-fatal suicide attempts only (HR: 2•34; 95% CI: 2•02–2•71), demonstrating that approximately 57.1% of the excess risk of PTSD on suicide in adjusted model 1 was due to confounding by previous non-fatal suicide attempt.

In a univariable model, those with a PTSD diagnosis were at greater risk of suicide than those without (HR: 4•28; 95% CI: 3•70–4•94; Table 2). This association increased slightly after adjustment for socio-demographic characteristics (HR_adj1_: 5•36; 95% CI: 4•64–6•20), but attenuated after further adjustment for psychiatric conditions diagnosed before PTSD and parental SMI (HR_adj2_: 2•16; 95% CI: 1•86–2•50; Table 2 and Supplemental file, Table 3). Further examination of the reason for this attenuation (Fig. 1), suggested this was primarily attributable to a history of non-fatal suicide attempts prior to a PTSD diagnosis, but not to other previous psychiatric conditions. Across all three models, there was evidence that elevated suicide rates associated with PTSD were stronger in women than men (i.e. Women HR_adj2_: 2•61; 95% CI: 2•16–3•14; Men HR_adj2_: 1•67; 95% CI: 1•31–2•12; LRT *p* = 0•003; Table 2).

**Table 3:** Attributable impact of PTSD on risk of death by suicide in the population at risk.

	Total		
% (95% CI)	Men		
% (95% CI)	Women		
% (95% CI)			
PTSD			
*Population attributable risk fraction (%)*			
Unadjusted	2•3 (1•9–2•8)	1•1 (0•8–1•5)	5•9 (4•7–7•1)
Adjustment 1^1^	2•5 (2•0–2•9)	1•1 (0•8–1•5)	5•9 (4•7–7•1)
Adjustment 2^2^	1•6 (1•2–2•1)	0•7 (0•3–1•0)	3•8 (2•5–5•0)
*Attributable Fraction in the exposed (%)*			
Unadjusted	76•6 (73•0–79•7)	73•7 (66•7–79•3)	85•5 (82•5–87•9)
Adjustment 1^1^	81•3 (78•4–83•9)	74•6 (67•7–80•0)	85•3 (82•3–87•8)
Adjustment 2^2^	53•7 (46•1–60•2)	44•3 (28•8–56•5)	54•7 (37•2–55•2)
			
Broadly-defined PTSD^3^			
*Population attributable risk fraction (%)*			
Unadjusted	3•3 (2•8–3•7)	1•9 (1•5–2•4)	7•2 (5•9–8•5)
Adjustment 1^1^	3•4 (2•9–3•9)	1•9 (1•5–2•4)	7•2 (5•9–8•5)
Adjustment 2^2^	2•4 (1•9–3•0)	1•4 (0•9–1•8)	4•8 (3•4–6•1)
*Attributable Fraction in the exposed (%)*			
Unadjusted	79•7 (77•0–82•1)	80•0 (75•8–83•5)	86•3 (83•8–88•4)
Adjustment 1^1^	83•6 (81•4–85•6)	80•4 (76•2–83•8)	86•1 (83•5–88•2)
Adjustment 2^2^	59•9 (54•2–65•0)	58•4 (49•0–66•0)	56•9 (48•2–64•1)

PTSD: post-traumatic stress disorder; 95% CI: 95% confidence interval.

^1^ Adjustment 1: for current age, natal sex (except in stratified analyses), country of origin, population density and deprivation.

^2^ Adjustment 2: for variables listed in Adjustment 1, previous major depression or anxiety disorders, bipolar disorder, non-affective psychotic disorder, previous non-fatal suicide attempts and parental history of severe mental illness.

^3^ See first sensitivity analysis for definition.

In our fully-adjusted model, we estimated that up to 1•6% (95% CI: 1.2-2.1) of all suicides could be prevented in the Swedish population aged 14 to 44 years old if no one experienced PTSD, assuming causality (Table 3). This figure was higher for women (PAF: 3•8%; 95% CI: 2•5–5•0) than men (PAF: 0•7%; 95% CI: 0•3–1•0). If we could identify and remove all factors that increased suicide risk in people with PTSD (i.e. the AFe), we could prevent 53•7% (95% CI: 46•1–60•2) of all suicides in those aged 14 to 44 years old exposed to PTSD in Sweden (Table 3).

Examination of a log-log residual plot (Supplemental file, Fig. 1) and a Schoenfeld residuals test (*p* = 0•28) found no evidence of departure from proportionality for our main exposure variable of PTSD diagnosis in our fully-adjusted model.

### 3.1 Sensitivity analyses

In our first sensitivity analyses, use of a broader PTSD definition did not alter the interpretation of our findings in relation to suicide (i.e. HR_adj2_: 2•50; 95% CI: 2•19–2•84; Table 2), although there was weaker evidence of effect modification by sex in our final model (LRT *p* = 0•11). The PAF associated with a broader PTSD definition rose to 4•8% (95% CI: 3•4–6•1) and 1•4% (95% CI: 0•9–1•8) in women and men, respectively (Table 3), and the overall AFe rose to 59•9% (54•2–65•0). In our second sensitivity analyses, we observed similar patterns of association between PTSD and both death by suicide (ICD-10 × 60–84) and death by undetermined intent (Y10–34), when analysed separately (Supplemental file, Table 4). In our third sensitivity analyses, we excluded 1613 people diagnosed with PTSD in outpatient settings between 2001 and 2005 (7.2% of all PTSD cases); this did not alter the interpretation of our findings (Supplemental file, Table 5). Finally, in our last sensitivity analysis to consider competing risks for suicide, people diagnosed with PTSD were also more likely to die by other reasons than those without PTSD (0•7% vs, 0•5%), and less likely to emigrate from Sweden (1•6% vs. 9•1%; Χ^2^-test on 3 degrees of freedom *p*<0•0001; Supplemental file, Table 6). However, sub-hazard ratios from competing risk regressions results models were generally consistent with patterns observed in our main analyses (Supplemental file, Table 6), although there was weaker evidence of sex differences in suicide risk associated with PTSD in our final model (Adjustment 2; LRT *p* = 0•06).

## 4 Discussion

### 4.1 Main findings

In this nationwide study, we found longitudinal evidence that PTSD was associated with increased suicide rates, with individuals diagnosed with PTSD twice as likely to die by suicide than those without PTSD. There was robust evidence that this association was moderately stronger in women than men. These effects were independent of other previous psychiatric disorders, including a personal history of major depressive or anxiety disorders, severe mental illness and suicide attempt, and parental diagnosis of a severe mental illness. However, our adjusted estimates were attenuated primarily by previous non-fatal suicide attempts, and this may relate to the trauma of the index stressor. We quantified the potential population impact of PTSD on suicide, and assuming causality, estimated that up to 1.6% of deaths by suicide could be prevented in people aged 14 to 44 years in the general population (and up to 3.8% for women), equivalent to over 50% of all suicides in people with PTSD. Taken together, our results can be used to support clinical decision making in identifying those with PTSD at greater risk of suicide than the general population.

### 4.2 Strengths and limitations

We used a large population-based cohort of over 3•1 million people, followed for over 49•2 million person-years, with minimal loss to follow-up. This gave us the ability to detect precise patterns of suicide risk associated with PTSD, adjusting for several potential sociodemographic and clinical confounders. Our results were also robust to investigation of potential biases, including competing risks or misclassification of either our exposure (i.e. a narrower versus broader PTSD definition) or outcome (suicide versus deaths of undetermined intent).

The Swedish registers are known to be reliable for psychiatric research because they provide completed records of all psychiatric contacts from inpatient settings (1987 onwards) and outpatient settings (2001 onwards), with strong reliability of suicide classification (Tøllefsen et al., 2015) and the validity of PTSD diagnoses in the national patient register (A.C. Hollander et al., 2019). While our included sample differed on exposure and covariate data from the small proportion (0•5%) of people we excluded due to missing data, the effect of this potential bias on our results would have been minimal.

We could not exclude the possibility of residual confounding in our results. This may have been an issue for our measures of a previous history of depressive or anxiety disorders, which was restricted to major disorders identified and treated via secondary or emergency mental healthcare services in Sweden. The national patient register does not include diagnoses solely made in primary care, meaning we would have missed depressive and neurotic psychopathologies in the population. Nevertheless, when we controlled for a broad range of major personal and family psychopathologies, elevated rates of death by suicide remained independently associated with PTSD. We did not adjust for the role of drug or alcohol misuse as we presumed that these might lie on the causal pathway, but could not explore the effects of adding these to final models due to the lack of primary care substance use data.

In this study, we controlled for non-fatal suicide attempts (operationalised as an act of self-harm recorded at least 31 days before a recorded suicide death) that warranted clinical attention and occurred prior to a PTSD diagnosis. Our analyses (Fig. 1) showed that adjustment for this clinical variable attenuated the association between suicide and PTSD to a greater extent than any other sociodemographic or clinical variable. Although it is possible that this represented an over-adjustment, we felt it was an important clinical variable to take into account in this patient population. We restricted our measure of non-fatal suicide attempts occurring before a PTSD diagnosis (and hence not downstream of the PTSD diagnosis), but do not know the exact date on which PTSD symptoms first began. If PTSD symptomatology had led to suicide attempts precipitating a subsequent diagnosis of PTSD, our fully-adjusted model results (Adjustment 2, Table 2) may be conservative, and would imply that the true association between PTSD and suicide is closer to the fivefold estimates of increased risk obtained under adjusted model 1.

Using register data meant we were unable to investigate how the trauma associated with a PTSD diagnosis might influence its association with suicide. Neither ICD-9 nor ICD-10 classification systems distinguish complex PTSD (relating to multiple traumas) from PTSD linked to one index trauma. They also provide no information on the types of traumas precipitating PTSD. Given that the number of traumatic events in individuals with PTSD is believed to be a significant predictor of suicidal behaviours (Afzali et al., 2017), it is possible that different types and/or numbers of experiences of PTSD-related trauma may vary in terms of their association with death by suicide. Further research is needed to establish whether this is the case.

## 5 Results in the context of other studies

Two previous longitudinal studies in Denmark (Gradus et al., 2010, 2015) reported large effect sizes (odds ratio: 5•3; 95% CI 3•4–8•1,(Gradus et al., 2010)) (rate ratio: 13•0; 95% CI 4•3–42•0 (Gradus et al., 2015)) for PTSD on suicide after adjustment for some sociodemographic and baseline psychiatric conditions, although not previous non-fatal suicide attempts. The magnitude of these associations was similar to our results prior to adjustment for suicidal attempts (HR_adj2E_: 4•23; 95% CI: 3•66–4•89; Fig. 1). Our analyses suggest that up to 57% of the effect of PTSD on suicide risk may be attributable to prior suicide attempts. Our findings contrast with reports from several studies of US military populations, which have observed that people with PTSD are less likely to die by suicide following a diagnosis (Britton et al., 2017; Conner et al., 2014; Desai et al., 2008; Zivin et al., 2007). However, such studies may not generalise beyond these specific institutionalised settings, where the funding and support infrastructure is designed to identify and provide specialised treatment for PTSD (Conner et al., 2013). Some of these studies may have over-adjusted for comorbid psychiatric conditions on the causal pathway (Britton et al., 2017; Desai et al., 2008).

We found that the association between PTSD and death by suicide was stronger in women than men, as previously observed in a US study of veterans (Ilgen et al., 2010). Previous US work has found that childhood trauma predicted suicidal behaviours in women but not men (Weiss et al., 2016). Gender differences were also observed in a Swedish general population sample, in which it was found that specific traumas are characteristic of those precipitating PTSD in women (rape and other sexual assault) and men (combat experience), and that women with PTSD report significantly higher trauma intensity (the perceived distress associated with that trauma) than men with PTSD (Frans et al., 2005). We suggest that the nature of the traumas characteristically experienced by women subsequently diagnosed with PTSD and the level of distress they perceive in association with this trauma may contribute to a relatively higher risk of suicide, and that this may be compounded by stigma, shame, and legal processes. In our nationwide sample, crude *absolute* suicide rates were higher in men than women diagnosed with PTSD (i.e. 67•2 vs. 48•5 per 100,000 person-years; Table 2) but the *relative* risk was stronger in women than men, attributable to higher baseline suicide rates in men than women in the general population. These results underscore the need for clinical mental health services to recognise both absolute and relative risks of suicide in people with PTSD.

Various theories have been proposed for the mechanisms that may account for the association between PTSD and suicide (Johnson et al., 2008; O'Connor and Kirtley, 2018; Van Orden et al., 2005; Williams et al., 2005). These suggest that key contributors to the onset of suicidal ideation are a sense of defeat, perceptions of powerlessness or a failing struggle (P Gilbert, 2000), and through entrapment, the feeling of being unable to escape an unpleasant state or situation (Paul Gilbert and Allan, 1998). PTSD could plausibly lead to feelings of defeat and entrapment, through the sense that recurrent symptoms of intrusive thoughts, nightmares and flashbacks are inescapable (Panagioti et al., 2012). Individuals with PTSD may also experience heightened physiological reactions when reminded of their trauma (Bedi and Arora, 2007), which when combined with autonomic hyperarousal, result in maintenance of perceptions of ongoing threat, entrapment and defeat (Selaman et al., 2014). Various strands of empirical evidence appear to support a role for defeat, entrapment and hyperarousal. A study of Israeli men, for example, found that hyperarousal symptoms were associated with higher suicide risk (Ben-Ya'acov and Amir, 2004), while other studies have observed that individuals with PTSD reported elevated levels of defeat and entrapment (Panagioti et al., 2012), that defeat and entrapment mediated the relationship between PTSD symptoms and suicidal behaviour (Panagioti et al., 2013) and that these predicted changes in levels of suicidal ideation (Panagioti et al., 2015).

### 5.1 Clinical, policy and research implications

Our findings have implications for the clinical management of people with PTSD, who appear to be at approximately twice the risk of death by suicide than the general population. This figure is likely to be substantially higher in those with a history of previous non-fatal suicide attempts, and those who belong to other high-risk groups for suicide (Supplemental file, Table 3), including those with a personal or parental history of psychiatric problems, young adults (especially those aged 20 to 24 years in our study population), and people from more deprived communities. The short median time between a PTSD diagnosis and death by suicide of less than 2.5 years highlights the need to act quickly and, where appropriate, intensively to prevent suicide in this group.

Although we did not explore specific means of suicide in patients with PTSD, means restriction is the suicide prevention intervention with the strongest evidence for effectiveness (Zalsman et al., 2016), and would be an important consideration in this patient group, particularly where prescribing potentially toxic medications. Less evidence is available on the effectiveness of trauma-focused cognitive behaviour therapy (CBT) or other exposure therapies on suicidality (Cusack et al., 2016), as trial outcomes are typically based on symptom severity. Clinical guidelines on the management of PTSD, such as those produced by NICE (National Institue for Health and Care Excellence, 2018), should recognise the increased risk of suicide in patients with PTSD, and acknowledge the need for further clinical trials to establish the effectiveness and safety of interventions for PTSD with respect to suicide-related outcomes.

In terms of public mental health, the PAF estimates in this population suggest that PTSD would account for 0•6% of suicides in men and 3•5% in women. In comparison to PAF estimates for other known risk factors for suicide, such as self-harm (PAF: 7•2% (Lewis et al., 1997)) and childhood sexual abuse (PAF: 11•3% (McLafferty et al., 2018)), this estimate is comparatively low, but still warrants inclusion within suicide prevention strategies of a recommendation for effective treatments for PTSD. Furthermore, in high risk groups, such as those with PTSD, our data suggest that (assuming causality) over half of suicides could be prevented if we could successfully prevent, treat and manage PTSD. International suicide prevention strategies (World Health Organization, 2014) should be updated to reflect this in light of these consistent findings from population-based studies in two different countries.

## Contributions

AP developed the hypothesis and designed the protocol and statistical analysis plan with JK and VF. HD, CD, ACH and VF cleaned and coded data. VF managed the literature searches, with input from AP, and wrote the first draft of the manuscript. All authors contributed to and have approved the final manuscript.

## Role of the funding source

JK was supported by a Sir Henry Dale Fellowship (Wellcome Trust/Royal Society grant: 101,272/Z/13/Z). AP and JK are supported by the National Institute for Health Research (NIHR) University College London Hospital (UCLH) Biomedical Research Centre (BRC). CD is supported by the Swedish Research Council (grant: 523–2010–1052). The funders of the study had no role in study design, data collection, data analysis, data interpretation, or writing of the report. JK had full access to all the data in the study and AP had final responsibility for the decision to submit for publication.

## Declarations of Competing Interest

None of the authors have any conflicts of interest.

## Appendix Supplementary materials

Image, application 1
