# Supplementary material for: Suicide risk in people with post-traumatic stress disorder: A cohort study of 3.1 million people in Sweden
Source: J Affect Disord. 2021 Jan 15;279:609–16. doi: 10.1016/j.jad.2020.10.009 (PMC7758737; doi:10.1016/j.jad.2020.10.009)
Supplement: Supplementary file 1 [file mmc1.docx]

**Supplemental Materials**

**Supplemental Methods**

*Design & setting*

Data were taken from Psychiatry Sweden, a database of linked national registers, which included hospital admissions for psychiatric disorders since 1973 on people officially resident in Sweden from January 1932, identified though unique personal identity numbers and anonymised for research by Statistics Sweden. Psychiatry Sweden was original granted ethical approval to use linked register data collected until the end of 2011. It was later granted approval to extend register linkage and follow-up until the end of 2016. However, this extended approval did not allow for inclusion of new residents in Sweden (by birth or immigration) after 2011. Therefore, our cohort only includes immigrant participants who entered Sweden prior to the end of 2011. All participants in our cohort were followed until the end of 2016.

*Confounder definitions*

Previous psychiatric conditions included in- or out-patient diagnoses for major depression, anxiety disorders, non-affective psychotic disorders, bipolar disorder or previous non-fatal suicide attempts, diagnosed prior to a recorded diagnosis of PTSD (for codes, see Supplemental Table 1). For previous suicide attempts, we identified the last non-fatal suicide attempt in all participants, restricted to the last non-fatal suicide attempt before a PTSD diagnosis in this group. Based on clinical expertise (AP), we defined last “non-fatal” suicide attempt as the last recorded in- or out-patient diagnosis of suicide attempt (including those of undetermined intent) which did not result in death by suicide (or undetermined intent) within 31 days of the date of the suicide attempt.

Parental history of severe mental illness included non-affective psychoses or bipolar disorder (Supplemental Table 1) in the biological parents, linked via the multi-generation register.

Neighbourhood-level variables were based on population density (people per square kilometre) and multiple deprivation in each participant’s residential neighbourhood (based on “Small Area Marketing Statistics” [SAMS] areas) in their year of cohort entry. We categorised each neighbourhood variable into quintiles.

Neighbourhoods were defined by “Small Area Marketing Statistics” [SAMS] areas, used in Sweden since 1982 to enumerate the population according to socially homogenous areas with a median estimated total population size in 2011 of 726 people (interquartile range [IQR]: 312-1378).^1^ Cohort participant neighbourhood-level covariates were linked to their SAMS area of residence at cohort entry (aged 14 years old or immigration to Sweden if later, up to the end of 2011) as recorded in the Register of the Total Population.

Neighbourhood deprivation was estimated using a multidomain index of deprivation at SAMS area level for each year of follow-up until 2011. The index comprised the sum of z-standardised scores of the proportion of the neighbourhood population in each SAMS who were unemployed, had an annual income below the national median, were in receipt of social benefits or who had been convicted of a criminal offence, as previously described.^1^ Data were linked from the Longitudinal Integration Database for Health Insurance and Labour Market Studies [LISA] and SAMS register to the Register of the Total Population. Bases for the four index measures were restricted to the adult-age population aged 16 years and above.

*Population attributable risk fraction [PAF] and attributable fraction in the exposed [AFe]*

We estimated the population attributable risk fraction and attributable fraction for those exposed to PTSD in our univariable, partially and fully-adjusted models using the user-written *punafcc* command in Stata.^2^ Briefly, following model fit, the mean between-scenario population HR (or “population unattributable fraction” [PUF]) is estimated based on two population scenarios; the real-world scenario as observed, and a hypothetical scenario where no one in the population is exposed to PTSD. The PUF is the proportion of the outcome, suicide, in the observed scenario that would remain in the hypothetical scenario if no one were exposed to PTSD; subtracting the PUF from one leads to an estimate of the PAF. This estimation method also allows the user to specify a subpopulation (here, those exposed to PTSD) to calculate the AFe.

*Competing risks regression*

We fitted Fine and Gray^3^ competing risks regressions to inspect the sensitivity of our models to the possibility that other deaths or emigration were competing risks for suicide in our data. We first tabulated the reason for cohort exit by PTSD status, and then fitted Fine & Gray competing risks regressions for our main exposure (PTSD) on suicide risk. We reported sub-hazard ratios from these models for the risk of suicide due to PTSD, overall and for men and women separately, after accounting for the possible effects of emigration and other deaths as competing events for our outcome.

**Supplemental Results**

Those with missing data (N=16,435; 0·5%) were excluded because no residential neighbourhood “SAMS” identifier was recorded at cohort entry (N=11,295; 68·7%), a SAMS identifier was present but could not be linked to neighbourhood covariate data (N=4,628; 28·2%) or due to unknown birth region (N=512; 3·1%).

**Supplemental references**

1 Lewis G, Dykxhoorn J, Karlsson H, *et al.* Assessment of the Role of IQ in Associations between Population Density and Deprivation and Nonaffective Psychosis. *JAMA Psychiatry* 2020; published online March 11. DOI:10.1001/jamapsychiatry.2020.0103.

2 Newson RB. Attributable and unattributable risks and fractions and other scenario comparisons. *Stata J* 2013; **13**: 672–98.

3 Fine JP, Gray RJ. A Proportional Hazards Model for the Subdistribution of a Competing Risk. *J Am Stat Assoc* 1999; **94**: 496–509.

**Supplemental Figure 1: Log-log plot to inspect proportional hazards assumption for the association between PTSD and suicide**

**
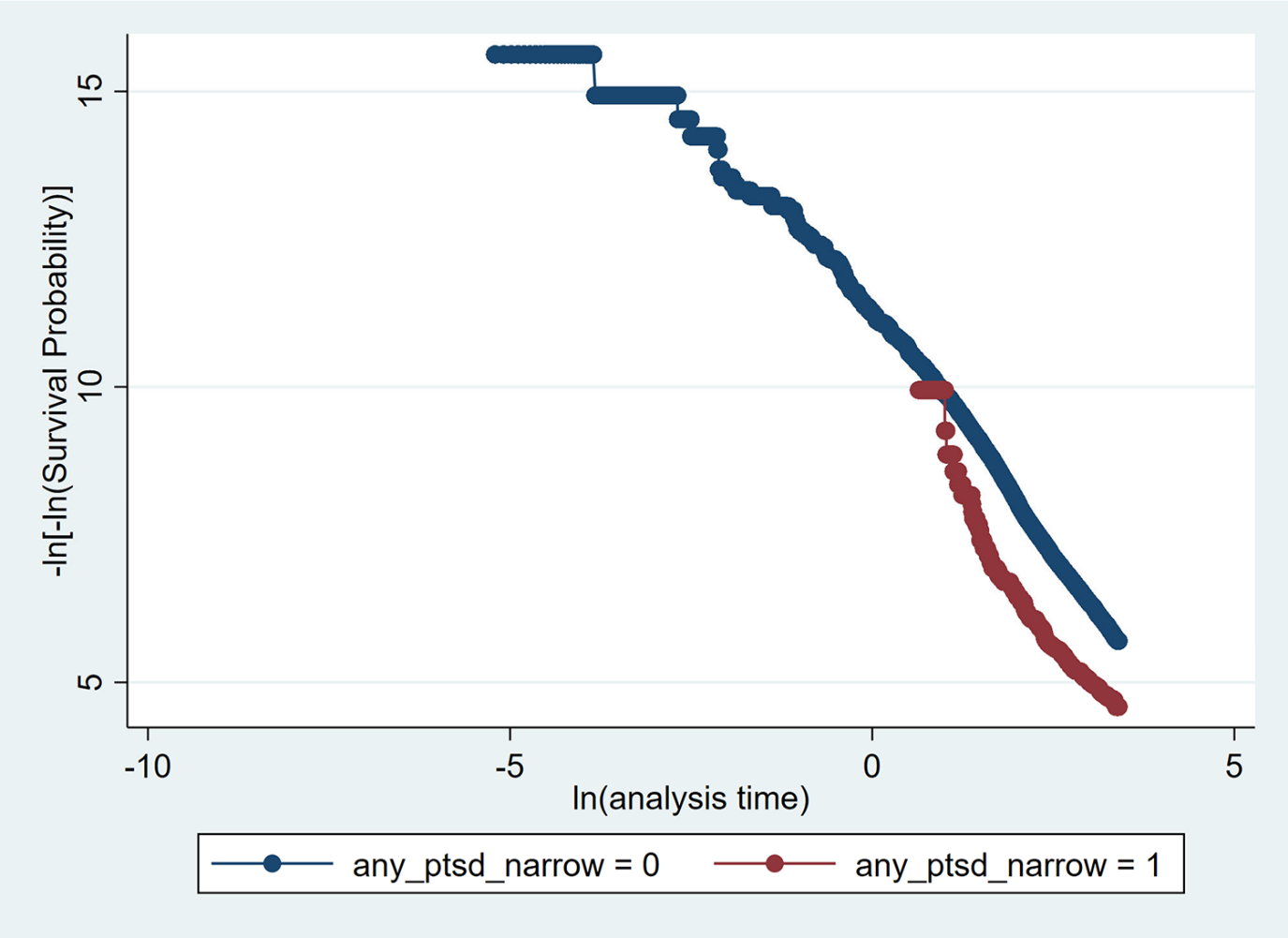
**

Legend: A proportional hazards test of Schoenfeld residuals of adjusted model 2 (see Table 2) found no evidence of non-proportional hazards for the association between PTSD and suicide (p=0·28), confirmed by approximately parallel lines from the log-log plot

**Supplemental Table 1: Diagnostic codes used to record major psychiatric conditions and parental severe mental illness**

|  | ICD-9 code | ICD-10 code |
| --- | --- | --- |
| PTSD^1^ | 309B | F43·1 |
| PTSD (broad, sensitivity analyses)^1^ | 309A,B,X | F43·1 |
| Major depressive disorders | 296C-D, 311 | F32-33 |
| Anxiety-related disorders | 300 | F40-41 |
| Non-affective psychoses | 295, 297, 298 | F20-29 |
| Bipolar disorder | 296A-B, E-H,X | F30-31 |
| Previous hospitalisation for non-fatal suicide attempts^2^ | E950-959, E980-989 | X60-84, Y10-34 |

^1^ The specific ICD-9 diagnostic code (309.81) for PTSD was not employed in Sweden. Instead, we used 309B “delayed stress reaction” as the closest proxy as our primary exposure variable. In a sensitivity analysis, we considered a broader ICD-9 PTSD classification, including the codes “acute stress reactions (309A)” and “crisis reactions, not otherwise specified” (309X) (see Tables 2 & 3)

^2^ Includes events of undetermined intent

**Supplemental Table 2: Sample differences according to missingness**

|  | Complete case sample  N (%) | Missing data  N (%) | Test (df); p-value^1^ |
| --- | --- | --- | --- |
| Total | 3,177,706 (99·5) | 16,435 (0·5) | - |
|  |  |  |  |
| Men | 1,626,697 (51·2) | 8,786 (53·5) | 33·7 (1); <0·0001 |
| Age at cohort exit (years) (median, IQR) | 30·5 (24·9-37·1) | 26·5 (21·7-33·0) | 55·4; <0·0001 |
| Birth region |  |  | 7·6x10^4^ (9); <0·0001 |
| Sweden | 2,524,627 (79·5) | 3,076 (19·3) |  |
| Finland | 21,886 (0·7) | 1,760 (11·1) |  |
| Other Nordic | 39,262 (1·2) | 2,916 (18·3) |  |
| Western & Southern Europe | 53,937 (1·7) | 1,350 (8·5) |  |
| Eastern Europe & Russia | 147,470 (4·6) | 2,182 (13·7) |  |
| Asia & Oceania | 120,726 (3·8) | 1,802 (11·3) |  |
| Middle East & North Africa | 156,427 (4·9) | 1,589 (10·0) |  |
| Sub-Saharan Africa | 62,752 (2·0) | 688 (4·3) |  |
| North America | 18,160 (0·6) | 294 (1·9) |  |
| South America | 32,459 (1·0) | 258 (1·6) |  |
| *Unknown*^2^ | - | *520* (-) |  |
| Population density (ppkm2) (median, IQR) | 754·1 (70·5-2,624·0) | 516·0 (59·1-5,522·5) | -1·4; 0·15 |
| Deprivation index (median, IQR) | -0·4 (-1·4-1·0) | 1·5 (0·0-3·8) | -16·8; <0·0001 |
|  |  |  |  |
| Deaths by suicide | 6,319 (0·2) | 24 (0·2) | 2·3 (1); 0·13 |
| Deaths, total all causes | 21,474 (0·7) | 119 (0·7) | 0·6 (1); 0·45 |
|  |  |  |  |
| PTSD | 22,361 (0·7) | 140 (0·9) | 5·1 (1); 0·02 |
| Previous diagnosis of major depression or anxiety | 297,612 (9·4) | 440 (2·7) | 864·5 (1); <0·0001 |
| Previous bipolar disorder diagnosis | 28,432 (0·9) | 41 (0·3) | 77·1 (1); <0·0001 |
| Previous non-affective psychosis diagnosis | 24,347 (0·8) | 74 (0·4) | 22·3 (1); <0·0001 |
| Previous non-fatal suicide attempts | 102,941 (3·2) | 205 (1·3) | 207·6 (1); <0·0001 |
| Parental history of SMI^3^ | 99,554 (3·1) | 225 (1·4) | 168·1 (1); <0·0001 |
|  |  |  |  |
| Reason for cohort exit |  |  | 5·9x10^4^ (3); <0·0001 |
| No censorship | 2,869,071 (90·3) | 5,771 (35·1) |  |
| Emigration | 287,161 (9·0) | 10,545 (64·2) |  |
| Suicide | 6,319 (0·2) | 24 (0·2) |  |
| Other death | 15,155 (0·5) | 95 (0·6) |  |

df: degrees of freedom; IQR: interquartile range; ppkm2: people per square kilometre

^1^ Χ^2^-test for binary & categorical data on *i* degrees of freedom (df); Mann-Whitney U-test for continuous data

^2^ Row not included in test

^3^ Includes non-affective psychosis and bipolar disorder

**Supplemental Table 3: Full model parameters from Cox regression models of the association between suicide and PTSD**

|  | Hazard ratios (95% CI) | | |
| --- | --- | --- | --- |
|  | **Unadjusted** | **Adjustment 1^1^** | **Adjustment 2^2^** |
| PTSD^3^ |  |  |  |
| Male | 3·81 (3·00-4·83) | 3·96 (3·12-5·03) | 1·67 (1·31-2·12) |
| Female | 6·85 (5·70-8·23) | 6·74 (5·61-8·09) | 2·61 (2·16-3·14) |
| Current age (years) |  |  |  |
| 14-19 | 1 | 1 | 1 |
| 20-24 | 1·35 (1·07-1·69) | 1·28 (1·01-1·62) | 1·57 (1·24-2·00) |
| 25-29 | 0·69 (0·54-0·89) | 0·65 (0·50-0·84) | 0·93 (0·71-1·21) |
| 30-34 | 0·52 (0·39-0·68) | 0·48 (0·36-0·64) | 0·78 (0·58-1·10) |
| 35-39 | 0·39 (0·28-0·55) | 0·36 (0·25-0·52) | 0·65 (0·45-0·94) |
| 40-44 | 0·59 (0·37-0·94) | 0·54 (0·33-0·89) | 1·06 (0·65-1·73) |
| Birth region |  |  |  |
| Sweden | 1 | 1 | 1 |
| Finland | 2·50 (1·92-3·25) | 2·73 (2·09-3·56) | 2·21 (1·69-2·88) |
| Other Nordic countries | 1·07 (0·75-1·53) | 1·22 (0·85-1·75) | 1·23 (0·86-1·77) |
| Western & Southern Europe | 0·65 (0·45-0·92) | 0·78 (0·55-1·12) | 0·80 (0·56-1·15) |
| Eastern Europe & Russia | 0·75 (0·64-0·88) | 0·76 (0·64-0·90) | 0·82 (0·70-0·97) |
| Asia & Oceania | 1·07 (0·91-1·26) | 1·24 (1·05-1·48) | 1·26 (1·07-1·49) |
| Middle East & North Africa | 0·69 (0·58-0·81) | 0·65 (0·54-0·77) | 0·67 (0·56-0·79) |
| Sub-Saharan Africa | 0·84 (0·65-1·09) | 0·86 (0·66-1·11) | 0·92 (0·71-1·19) |
| North America | 0·98 (0·62-1·55) | 1·09 (0·68-1·73) | 1·02 (0·64-1·62) |
| South America | 1·29 (1·03-1·61) | 1·22 (0·98-1·53) | 1·06 (0·85-1·33) |
| Population density (ppkm^2^) (quintiles) |  |  |  |
| 1 (most rural) | 1 | 1 | 1 |
| 2 | 0·95 (0·88-1·02) | 1·02 (0·94-1·10) | 0·97 (0·90-1·05) |
| 3 | 0·92 (0·85-0·99) | 1·02 (0·94-1·10) | 0·95 (0·88-1·03) |
| 4 | 1·00 (0·92-1·07) | 1·08 (1·00-1·69) | 0·99 (0·91-1·07) |
| 5 (most urban) | 1·09 (1·01-1·18) | 1·09 (1·00-1·19) | 0·97 (0·89-1·05) |
| Deprivation index (quintiles) |  |  |  |
| 1 (most affluent) | 1 | 1 | 1 |
| 2 | 1·10 (1·01-1·19) | 1·12 (1·03-1·21) | 1·07 (0·98-1·16) |
| 3 | 1·19 (1·10-1·29) | 1·22 (1·12-1·32) | 1·13 (1·04-1·23) |
| 4 | 1·39 (1·28-1·50) | 1·42 (1·31-1·54) | 1·28 (1·18-1·39) |
| 5 (most deprived) | 1·44 (1·33-1·56) | 1·55 (1·42-1·69) | 1·33 (1·22-1·45) |
| Previous diagnosis of major depression or anxiety | 5·41 (5·14-5·69) | - | 3·28 (3·09-3·48) |
| Previous bipolar disorder diagnosis | 5·45 (4·90-6·06) | - | 1·27 (1·14-1·42) |
| Previous non-affective psychosis diagnosis | 12·11 (11·17-13·14) | - | 2·96 (2·71-3·23) |
| Previous non-fatal suicide attempts | 10·08 (9·54-10·66) | - | 4·94 (4·63-5·27) |
| Parental history of SMI | 2·84 (2·61-3·09) | - | 1·84 (1·68-2·01) |

PTSD: post-traumatic stress disorder; 95% CI: 95% confidence intervals; ppkm^2^: people per square kilometre; SMI: severe mental illness

^1^ Adjusted for current age, natal sex (except in stratified analyses), country of origin, population density and deprivation

^2^ Adjusted for variables listed in Adjustment 1, previous major depression or anxiety disorders, bipolar disorder, non-affective psychotic disorder, previous non-fatal suicide attempts and parental history of severe mental illness

^3^ As reported in Table 2; hazard ratios for main effect of suicide in men vs. women not reported in presence of statistically significant interaction between natal sex and PTSD.

**Supplemental Table 4: Sensitivity analyses for suicide (X60-84) versus deaths by undetermined intent (Y10-34) associated with PTSD**

|  | Cases | Non-cases | Crude mortality rate^2^ | | Hazard ratios (95% CI) | | |
| --- | --- | --- | --- | --- | --- | --- | --- |
|  | **N (%)^1^** | **N (%)^1^** | **Rate** | **95% CI** | **Unadjusted** | **Adjustment 1^3^** | **Adjustment 2^4^** |
| Suicide (X60-84) |  |  |  |  |  |  |  |
| *PTSD exposure* |  |  |  |  |  |  |  |
| No | 4,745 (0·15) | 3,150,600 (99·85) | 9·7 | (9·4-10·0) | 1 | 1 | 1 |
| Yes | 150 (0·67) | 22,211 (99·33) | 42·1 | (35·9-49·4) | 4·31 (3·66-5·07) | 5·37 (4·56-6·33) | 2·26 (1·91-2·67) |
| Men | 53 (0·80) | 6,612 (99·20) | 51·6 | (39·4-67·5) | 3·84 (2·93-5·04) | 4·05 (3·08-5·32) | 1·79 (1·36-2·35) |
| Women | 97 (0·62) | 15,599 (99·38) | 38·3 | (31·4-46·7) | 6·63 (5·39-8·14) | 6·56 (5·34-8·07) | 2·66 (2·16-3·27) |
| LRT test *Χ*^2^ for interaction (df); p-value |  |  |  |  | 10·3 (1); 0·001 | 8·0 (1); 0·005 | 5·4 (1); 0·02 |
|  |  |  |  |  |  |  |  |
| Undetermined intent (Y10-34) |  |  |  |  |  |  |  |
| *PTSD exposure* |  |  |  |  |  |  |  |
| No | 1,382 (0·04) | 3,153,963 (99·96) | 2·8 | (2·7-3·0) | 1 | 1 | 1 |
| Yes | 42 (0·19) | 22,319 (99·81) | 11·8 | (8·7-16·0) | 4·16 (3·06-5·66) | 5·32 (3·90-7·26) | 1·87 (1·37-2·57) |
| Men | 16 (0·24) | 6,649 (99·76) | 15·6 | (9·6-25·5) | 3·70 (2·26-6·07) | 3·65 (2·26-6·00) | 1·33 (0·81-2·19) |
| Women | 26 (0·17) | 15,670 (99·83) | 10·3 | (7·0-15·1) | 7·83 (5·25-11·68) | 7·48 (5·01-11·17) | 2·53 (1·69-3·79) |
| LRT test Χ^2^ for interaction (df); p-value |  |  |  |  | 5·5 (1); 0·02 | 5·0 (1); 0·02 | 4·0 (1); 0·05 |

LRT: likelihood ratio test; df: degrees of freedom; PTSD: post-traumatic stress disorder; 95% CI: 95% confidence intervals

^1^ Row percentages

^2^ per 100,000 person-years

^3^ Adjusted for current age, natal sex (except in stratified analyses), country of origin, population density and deprivation

^4^ Adjusted for variables listed in Adjustment 1, previous major depression or anxiety disorders, bipolar disorder, non-affective psychotic disorder, previous non-fatal suicide attempts and parental history of severe mental illness

**Supplemental Table 5: Sensitivity analyses excluding people diagnosed with PTSD in outpatient settings between 2001-2005^1^**

|  | Crude mortality rate | | | Hazard ratios (95% CI) | | | |
| --- | --- | --- | --- | --- | --- | --- | --- |
| *Original analyses (from Table 2)* | **Rate** | **95% CI** | **Unadjusted** | | **Adjustment 1^2^** | **Adjustment 2^3^** |  |
| PTSD (from Table 2) |  |  |  | |  |  |  |
| No | 12·6 | (12·3-12·9) | 1 | | 1 | 1 |  |
| Yes | 53·9 | (46·8-62·1) | 4·28 (3·70-4·94) | | 5·36 (4·64-6·20) | 2·16 (1·86-2·50) |  |
| Men | 67·2 | (53·1-85·1) | 3·81 (3·00-4·83) | | 3·96 (3·12-5·03) | 1·67 (1·31-2·12) |  |
| Women | 48·5 | (40·6-57·9) | 6·85 (5·70-8·23) | | 6·74 (5·61-8·09) | 2·61 (2·16-3·14) |  |
| LRT test *Χ*^2^ for interaction (df); p-value |  |  | 15·3 (1); 0·0001 | | 12·5 (1); 0·0004 | 8·8 (1); 0·003 |  |
|  |  |  |  | |  |  |  |
| *Sensitivity analyses (excluding N=1,613)^1^* |  |  |  | |  |  |  |
| PTSD |  |  |  | |  |  |  |
| No | 12·6 | (12·3-12·9) | 1 | | 1 | 1 |  |
| Yes | 53·6 | (46·3-62·2) | 4·27 (3·68-4·96) | | 5·37 (4·62-6·25) | 2·10 (1·80-2·44) |  |
| Men | 63·9 | (49·5-82·5) | 3·64 (2·81-4·70) | | 3·78 (2·92-4·89) | 1·53 (1·18-1·98) |  |
| Women | 49·6 | (41·3-59·5) | 7·03 (5·82-8·48) | | 6·89 (5·71-8·32) | 2·60 (2·15-3·15) |  |
| LRT test Χ^2^ for interaction (df); p-value |  |  | 17·3 (1); 0·0001 | | 14·4 (1); 0·0002 | 11·2 (1); 0·0008 |  |

^1^ Outpatient coverage in Sweden was incomplete from 2001-5. We excluded 1,613 people diagnosed with PTSD in outpatient settings during this time (7.2% of all people exposed to PTSD in our cohort) and re-ran our models to inspect any potential biases introduced by including these participants in our main analyses. Results were minimally altered.

^2^ Adjusted for current age, natal sex (except in stratified analyses), country of origin, population density and deprivation

^3^ Adjusted for variables listed in Adjustment 1, previous major depression or anxiety disorders, bipolar disorder, non-affective psychotic disorder, previous non-fatal suicide attempts and parental history of severe mental illness

^4^ See first sensitivity analysis for definition

**Supplemental Table 6: Sensitivity analyses using a competing risks regression to model association between PTSD and suicide, taking into account death by other causes and emigration as potential competing risks**

|  | Censorship reason (N, %)^1^ | | | | Sub-hazard ratios (95% CI) | | |
| --- | --- | --- | --- | --- | --- | --- | --- |
|  | **Suicide^2^** | **Other deaths** | **Emigration** | **No censorship** | **Unadjusted** | **Adjustment 1^3^** | **Adjustment 2^4^** |
| PTSD |  |  |  |  |  |  |  |
| No | 6,127 (0·19) | 15,008 (0·48) | 286,797 (9·09) | 2,847,413 (90·24) | 1 | 1 | 1 |
| Yes | 192 (0·86) | 147 (0·66) | 364 (1·63) | 21,658 (96·86) | 4·40 (3·81-5·08) | 5·90 (5·10-6·81) | 2·17 (1·87-2·53) |
| Men | 69 (1·04) | 88 (1·32) | 126 (1·86) | 6,382 (95·75) | 3·78 (2·98-4·80) | 4·60 (3·63-5·83) | 1·83 (1·43-2·34) |
| Women | 123 (0·78) | 59 (0·38) | 238 (1·52) | 15,276 (97·32) | 7·17 (5·97-8·61) | 7·05 (5·87-8·47) | 2·44 (2·02-2·95) |
| LRT test Χ2 for interaction (df); p-value |  |  |  |  | 18·2 (1); p<0·0001 | 8·0 (1); p=0·005 | 3·6 (1); p=0·06 |

^1^ A Χ^2^-test on 3 degrees of freedom suggested strong differences (p<0·0001) in censorship reasons (i.e. potential competing risks) between those with and without a PTSD diagnosis. Those with PTSD were more likely to have died (by suicide or other causes) and less likely to have emigrated than those without a PTSD diagnosis

^2^ Includes death by suicide (X60-84) and undetermined intent (Y10-34)

^3^ Adjusted for current age, natal sex (except in stratified analyses), country of origin, population density and deprivation

^4^ Adjusted for variables listed in Adjustment 1, previous major depression or anxiety disorders, bipolar disorder, non-affective psychotic disorder, previous non-fatal suicide attempts and parental history of severe mental illness
